# Contrasting Effects of Two Lipid Cofactors of Prion Replication on the Conformation of the Prion Protein

**DOI:** 10.1371/journal.pone.0130283

**Published:** 2015-06-19

**Authors:** Saurabh Srivastava, Ilia V. Baskakov

**Affiliations:** Center for Biomedical Engineering and Technology, Department of Anatomy and Neurobiology, University of Maryland School of Medicine, Baltimore, MD, 21201, United States of America; Scuola Internazionale Superiore di Studi Avanzati, ITALY

## Abstract

Recent studies introduced two experimental protocols for converting full-length recombinant prion protein (rPrP) purified from *E*.*coli* into the infectious prion state (PrP^Sc^) with high infectivity titers. Both protocols employed protein misfolding cyclic amplification (PMCA) for generating PrP^Sc^ *de novo*, but used two different lipids, 1-palmitoyl-2-oleolyl-*sn*-glycero-3-phospho(1’-*rac*-glycerol) (POPG) or phosphatidylethanolamine (PE), as conversion cofactors. The current study compares the effect of POPG and PE on the physical properties of native, α-helical full-length mouse rPrP under the solvent conditions used for converting rPrP into PrP^Sc^. Surprisingly, the effects of POPG and PE on rPrP physical properties, including its conformation, thermodynamic stability, aggregation state and interaction with a lipid, were found to be remarkably different. PE was shown to have minimal, if any, effects on rPrP thermodynamic stability, cooperativity of unfolding, immediate solvent environment or aggregation state. In fact, little evidence indicates that PE interacts with rPrP directly. In contrast, POPG was found to bind to and induce dramatic changes in rPrP structure, including a loss of α-helical conformation and formation of large lipid-protein aggregates that were resistant to partially denaturing conditions. These results suggest that the mechanisms by which lipids assist conversion of rPrP into PrP^Sc^ might be fundamentally different for POPG and PE.

## Introduction

Prion diseases or transmissible spongiform encephalopathies represent a class of neurodegenerative disorders of humans and animals [1]. The key event underlying prion diseases involves the conformational change of the α-helical, native, cellular form of the prion protein (PrP^c^) into a β-sheet rich, aggregated, transmissible form (PrP^Sc^) [2]. PrP^Sc^ replicates its conformation autocatalytically via recruiting and converting PrP^c^ molecules. This process occurs in parallel with fragmentation of PrP^Sc^ aggregates that leads to multiplication of active centers of replication. Owing to the autocatalytic nature of PrP^Sc^ replication, PrP^Sc^ aggregates can spread from cell to cell or between individual animals or humans.

As evident from X-ray analysis, PrP^Sc^ exhibits a cross-β sheet conformation [3], a key molecular feature of amyloid structure. In the absence of cellular cofactors amyloid fibrils produced *in vitro*, using highly pure recombinant PrP (rPrP), induced transmissible prion diseases in animals with new strain-specific features [4–10]. However, fibril preparations displayed limited infectivity in animal bioassays [11]. As judged from X-ray diffraction, rPrP fibrils and PrP^Sc^ were shown to have substantially different structures [12, 13] that could explain the apparently low infectivity of rPrP amyloid preparations. Because PrP^C^ is expressed on the cell surface, lipid rafts were thought to play an important role in conversion of PrP^C^ into *bona fide* PrP^Sc^, with lipids acting as potential cofactors [14–16]. Indeed, earlier studies illustrated that several lipids including 1-palmitoyl-2-oleoyl-*sn*-glycero-3-phosphoserine, 1-palmitoyl-2-oleoyl-*sn*-glycero-3-phosphocholine, and 1-palmitoyl-2-oleolyl-*sn*-glycero-3-phospho(1’-*rac*-glycerol) (POPG) could either unfold or induce conformational change in rPrP toward aggregated, proteinase K-resistant, β-sheet rich structures [17–23].

In the last few years two experimental protocols were developed in which rPrP was successfully converted into PrP^Sc^ with high infectivity titers [24, 25]. Both of these protocols employed protein misfolding cyclic amplification (PMCA) for generating PrP^Sc^ *de novo* and used lipids as conversion cofactors. PMCA is a technique developed by Soto and colleagues that consists of alternating cycles of sonication and incubation [26]. Conventional PMCA uses normal brain homogenate as a source of PrP^C^. However, Ma and colleagues showed that highly infectious PrP^Sc^ could be produced in PMCA using rPrP in the absence of normal brain homogenate, a complex and ill-defined mixture, but only in the presence of the anionic phospholipid POPG and total liver RNA [24]. Subsequently, Supattapone and colleagues showed that phosphatidylethanolamine (PE) can be used as a sole cofactor for generating prions with high infectivity titer *in vitro* [25, 27]. PE was found to be essential and sufficient for replicating PrP^Sc^ in PMCA that employs rPrP as a substrate [27].

The success of two different lipid-based protocols for generating highly infectious PrP^Sc^ from rPrP suggested that lipids alone or in combination with nucleic acids guide rPrP in the acquisition of infectious conformations. However, the mechanism of lipid-assisted conversion of rPrP is not known. It is also not clear whether lipid-assisted conversion follows a common mechanism when different lipids are used. In the current study we compared the effect of POPG and PE on the physical properties of full-length mouse rPrP under the solvent conditions used for converting rPrP into PrP^Sc^ [24, 25]. POPG and PE were found to have remarkably different effects on rPrP physical properties including its conformation, stability, aggregation state and interaction with a lipid. PE is a native lipid to mammalian cell membranes and harbored the native-like conformation of rPrP with little, if any, effects on its thermodynamic stability, cooperativity of unfolding, immediate solvent environment or aggregation state. In contrast, POPG, an anionic hydrophobic lipid that is less abundant in mammalian cells induced dramatic changes in protein structure. POPG was found to interact with rPrP directly that led to a loss of α-helical structure and formation of large lipid-protein aggregates that were resistant to partially denaturing conditions. The current study suggests that the mechanisms by which two lipids assist prion replication appears to be fundamentally different for POPG and PE.

## Materials and Methods

### Protein expression and purification

Full length mouse rPrP encompassing residues 23–231 was expressed and purified as previously described [28]. The purity of the final rPrP preparation was confirmed by sodium dodecyl sulfate polyacrylamide gel electrophoresis (SDS–PAGE) followed by silver staining and electrospray mass spectrometry to be a single species with an intact disulfide bond. Ten milligrams of 99.5+ % pure rPrP was obtained per liter of culture.

### Lipid preparations

Stock solutions of 25 mg/ml PE (Avanti polar lipids, Cat # 840022C) or POPG (Avanti polar lipids, Cat # 840457C) were prepared in chloroform-methanol (1:3) and stored at -20°C. For vesicle preparation, lipid solutions were diluted 10-fold in chloroform-methanol (1:3) and dried under nitrogen flow to form homogenous lipid films. These films were dried further overnight in nitrogen chamber to remove traces of organic solvents. Dried lipid films were hydrated with 0.05% Triton X-100 prepared in triple distilled water, then vortexed, and the resulting lipid suspensions were sonicated in a bath sonicator (Branson 2510, Branson Ultrasonics, Danbury, CT) until a clear suspension was obtained. Lipid preparations were kept under nitrogen and used fresh to avoid precipitation and oxidation of lipids.

rPrP-lipid mixtures were prepared as previously described [29]. Briefly, rPrP was diluted in triple distilled water, filtered with 0.22 μ filters (Millex-GV, Merck), then Triton X-100 added to a final concentration of 0.05%. Afterwards, Tris (1 M, pH 7.5), NaCl (5 M) and EDTA (0.5 M) were added to the final concentrations 20 mM Tris, 135 mM NaCl and 2 mM EDTA and the Triton X-100 was adjusted to 0.05%. The final concentration of Triton X-100 was maintained at 0.05% regardless of the presence or absence of lipids. The concentration of Triton X-100 in the current study was lower than those reported in the protocols on POPG- or PE-assisted conversions (Table 1), because concentrations of Triton X-100 above 0.05% were incompatible with the CD measurements due to high light scattering.

**Table 1:** Solvent conditions used in two PMCA protocols for converting rPrP into PrP^Sc^.

	Protocol with POPG [29]	Protocol with PE [25, 27]	Solvent conditions used in the current work
Buffer	10 mM Tris, pH 7.5	20 mM Tris, pH 7.5	20 mM Tris, pH 7.5
NaCl	150 mM	135 mM	135 mM
EDTA	1 mM	5 mM	2 mM
Detergent	0.25% Triton	0.15% Triton	0.05% Triton
rPrP	1.8 μM	0.26 μM	variable
Phospholipids	0.029 mM POPG	2.5 mM PE	variable

### Circular dichroism, thermal denaturation and data analysis

CD spectra of rPrP (5μM) were recorded in a 0.1 cm cuvette with a J-810 CD spectrometer (Jasco, Easton, MD), scanning at 20 nm/min, with a band width of 1 nm and data spacing of 0.5 nm. Each spectrum represents an average of two individual scans after subtracting the background spectra.

The thermal denaturations were recorded in a 0.1 cm cuvette with a J-810 CD spectrometer interfaced with a temperature control unit at 222 nm with a bandwidth of 1 nm. The samples were equilibrated at 20°C for 15 min following by increase of temperature with a constant rate of 1°C/min.

The data on dependence of mean residue ellipticity (Θ) on temperature (t) at 222 nm were fitted using a non-linear least-square fitting to the following equation assuming a two-state unfolding transition as previously described [30]:

$$\theta =\frac{{\text{exp}}^{\left(\left(\frac{H}{R*\left(t+T{}^{\circ}\right)}\right)*(\left(\frac{t+T{}^{\circ}}{Tm+T{}^{\circ}}\right)-1)\right)}}{1+exp^{\left(\left(\frac{H}{R*\left(t+T{}^{\circ}\right)}\right)*(\left(\frac{t+T{}^{\circ}}{Tm+T{}^{\circ}}\right)-1)\right)}}*(\left(n-\left(N*t\right)\right)-\left(u+\left(U*t\right)\right)+\left(u+\left(U*t\right)\right))$$

Because the denaturations of rPrP were not reversible, apparent melting temperatures are reported. The following fitting parameters were used in the equation: *Tm* is apparent melting temperature (°C), *H* is the apparent enthalpy of unfolding, *N* and *U* are the temperature dependence of the mean residue ellipticity for the folded and unfolded states, respectively, and *n* and *u* are the mean residue ellipticity approximated to 0°C for the folded and unfolded states, respectively; R = 1.987 cal/K*mol and T° = 273.15 K.

### Dynamic light scattering

Light scattering data were recorded in a 12 ul DynaPro quartz cuvette using a Protein Solutions DynaPro instrument (Wyatt Technology, Santa Barbara, CA). All solutions used to prepare the rPrP-lipid mixtures were filtered using 0.22 μ filters (Millex-GV, Merck). Each sample was measured in at least two independent acquisition sets with 20 or more independent data acquisition points collected for each set. Experimental data were analyzed using Dynamics V6 software.

### Tryptophan fluorescence spectroscopy

Tryptophan emission spectra were collected for rPrP (10 μM) alone or rPrP in the presence of 20 μM PE or 20 μM POPG using 0.4-cm rectangular cuvettes in a Fluoro-Max-3 fluorimeter (Jobin Yvon, Edison, NJ) with an excitation at 295 nm. The excitation and emission slits were set at 5 and 3 nm, respectively. To correct for a possible light scattering effects of lipids, the spectra of PE or POPG were subtracted from the corresponding spectra of rPrP/lipid mixtures. All samples were prepared in 20 mM Tris, pH 7.5, 135 mM NaCl, and 2 mM EDTA and 0.05% Triton. Deconvolution of emission spectra was performed by PeakFit software (version 4.12) using automated Gaussian fitting option. The r^2^ values were >0.99 for all fitting curves.

### Sucrose density gradient ultracentrifugation

rPrP (10μM) was incubated alone or with 500 μM PE or POPG in 20 mM Tris pH 7.5, 135 mM NaCl, 2m M EDTA, 0.05% Triton-X100 for 16 hours at room temperature. Then samples were adjusted to 40% (w/v) sucrose to make a final volume of 800 ul. This layer was over-laid with 3 ml of 30% sucrose, followed by 1 ml of 5% sucrose solutions to create a discontinuous sucrose gradient. Sucrose solutions were prepared in 20 mM Tris pH 7.5, 135 mM NaCl, 2 mM EDTA at room temperature. After centrifugation at 140,000g using Beckman coulter SW 55 Ti rotor at 4°C for 16 hrs, 10 fractions of 0.5 ml each were collected from top to bottom. For detecting rPrP in these fractions dot blots were performed according to the following procedure. 10 ul from each fraction was added to 10 ul of 2X-sample buffer (125 mM Tris pH 6.8, 2% SDS), gently vortexed, and then 5 ul from each sample was spotted on PVDF membrane without any denaturing steps. Once spots were dried, PVDF membranes were incubated with 2% BSA and probed with SAF-84 antibody. Each sucrose density gradient centrifugation experiment was repeated twice, and in experiment the samples were prepared in duplicates.

### Gel electrophoresis

rPrP was incubated with 50, 250 or 500 μM PE or POPG at room temperature for 16 hours in 20 mM Tris pH 7.5, 135 mM NaCl, 2 mM EDTA and 0.05% Triton. 2X loading buffer (125 mM Tris pH 6.8, 40% Glycerol, 0.02% Bromophenol blue) was added and samples were loaded in NuPAGE 12% Bis-Tris minigels (Novex, Life Technology) without temperature denaturation. Electrophoresis was performed in 1X MES-SDS running buffer (Novex); gels were stained with coomassie blue.

To assess the oligomerization state of rPrP in sucrose density gradient fractions, 10 ul of each fraction was incubated with 2X-sample buffer (125 mM Tris pH 6.8, 40% glycerol, 0.02% Bromophenol blue) for 10 min at room temperature. 10 ul of each fraction was loaded to NuPAGE 12% Bis-Tris minigels (Novex) without temperature denaturation. Electrophoresis was performed in 1X MES-SDS running buffer (Novex), and gels were stained with silver.

## Results

### rPrP is aggregated under the solvent conditions used in PMCA

**Fig 1:**
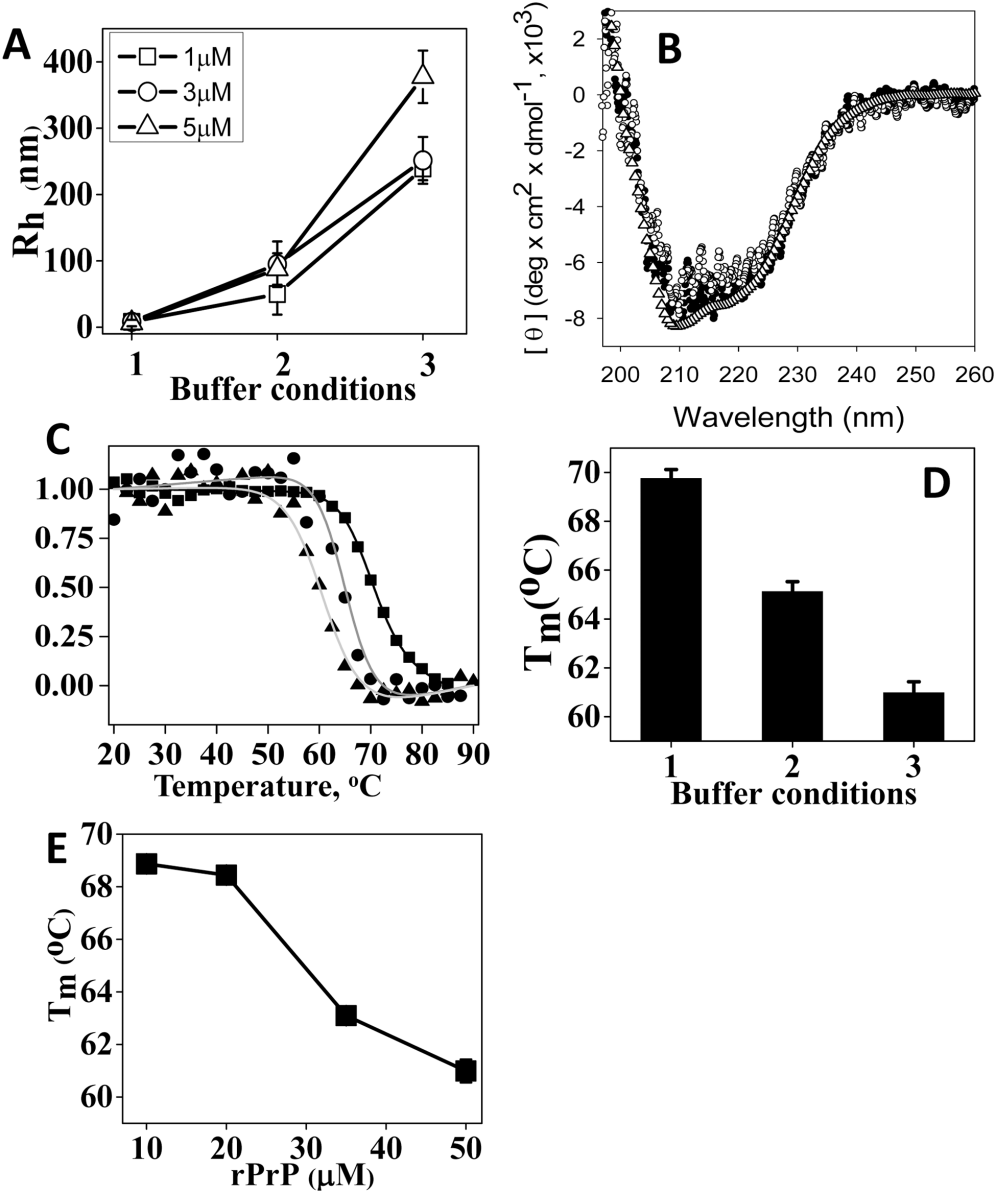
Analysis of rPrP physical properties under solvent conditions used for PMCA. (A) Analysis of hydrodynamic radius of rPrP at concentration 1 μM, 3 μM or 5 μM as indicate in 10 mM Na-acetate buffer, pH 5 (buffer condition # 1), 10 mM Na-acetate buffer, pH 5, 135 mM NaCl, 2 mM EDTA (condition # 2), or 20 mM Tris-HCl buffer, pH 7.5, 135 mM NaCl, 2 mM EDTA (condition # 3). Error bars represent percentage polydispersity for each sample (B) Far UV spectra of rPrP (5 μM) collected in 10 mM Na-acetate buffer, pH 5 (buffer condition # 1, open triangles), 20 mM Tris-HCl buffer, pH 7.5 in the absence (solid circle) or presence of 135 mM NaCl (open circle). (C) Temperature-induced unfolding of rPrP monitored by a circular dichroism at 222 nm and conducted in buffer condition # 1 (square), # 2 (circle), or # 3 (triangle). The solid lines represent the nonlinear least-square fit of the data to a two-state unfolding model. (D) The apparent melting temperatures (Tm) of rPrP denaturation measured in three buffer conditions specified in the panel A. (D) (E) Dependence of apparent melting temperature (Tm) on rPrP concentration. The temperature-induced denaturation of rPrP was conducted in 20mM Tris-HCl buffer, pH 7.5, 135 mM NaCl, 2mM EDTA and monitored by a circular dichroism at 222 nm.

Prior to examining the interaction of POPG or PE with rPrP, we tested whether the solvent conditions employed by the two protocols affected rPrP properties. Both protocols used Tris-HCl buffer pH 7.5 with slightly different concentrations of salt, EDTA and Triton (Table 1). In agreement with the previous studies [31], rPrP was found to be highly soluble in 10 mM Na-acetate buffer pH 5.0. It showed a hydrodynamic radius consistent with a monomer (Fig 1A), predominantly α-helical secondary structure as probed by CD (Fig 1B), and highly cooperative unfolding transition in temperature-induced denaturation with the apparent melting temperature (Tm) 69.8°C (Fig 1C and 1D). Supplementing Na-acetate buffer, pH 5.0 with 135 mM NaCl and 2mM EDTA induced considerable aggregation as judged by an increase in hydrodynamic radius (Fig 1A). While aggregated in the presence of salt, rPrP preserved α-helical conformation and highly cooperative unfolding in temperature denaturation, whereas the apparent Tm value decreased from 69.8°C to 65.1°C (Fig 1C and 1D). In 20 mM Tris-HCl buffer, pH 7.5, 135 mM NaCl, 2 mM EDTA the hydrodynamic radius of rPrP increased further, a sign of more pronounced aggregation (Fig 1A). Nevertheless, at pH 7.5 rPrP maintained α-helical conformation and cooperative unfolding (Fig 1B and 1C), although the apparent Tm value dropped to 61.0°C (Fig 1C). An increase in average size of rPrP aggregates and drop in apparent Tm value was also observed with an increase in protein concentration (Fig 1A and 1E). Nevertheless, the changes in solvent conditions such as addition of salt and an increase in pH had much more profound effect on hydrodynamic radius than protein concentration (Fig 1A).

In summary, the preliminary experiments showed that under solvent conditions used for PMCA protocols with POPG or PE, rPrP formed large aggregates of several hundred nm in hydrodynamic radius. Despite aggregation, rPrP preserved predominantly a α-helical conformation and native-like folding as evident by circular dichroism (CD) and cooperative unfolding transition. Nevertheless, α-helical folding domain showed substantially lower conformational stability relative to that observed in the absence of salt or pH 5.0.

### Contrasting effects of PE and POPG on rPrP conformation

**Fig 2:**
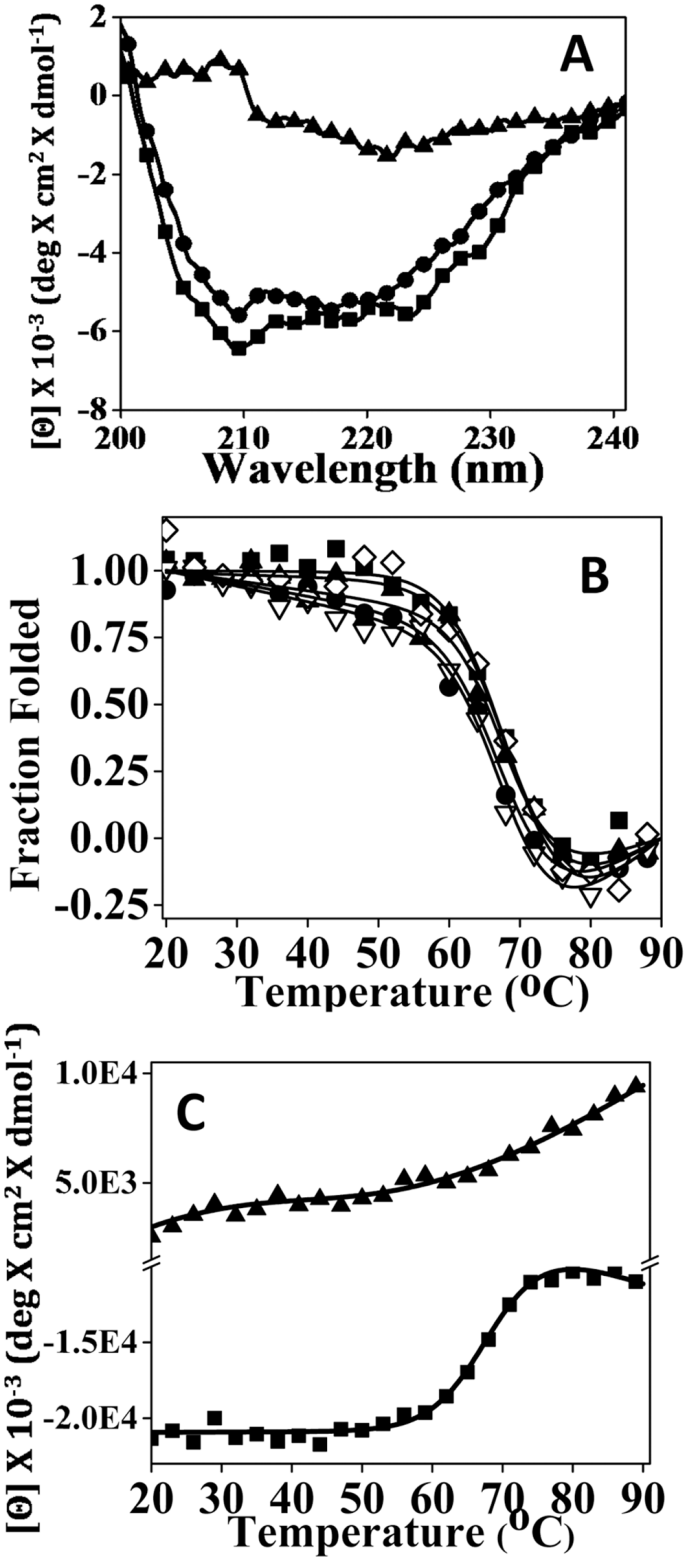
Effect of PE and POPG on conformation and stability of rPrP. (A) Far UV CD spectra of rPrP (5μM) alone (black squares) or in the presence of 10 μM PE (circle) or POPG (triangle). (B) Temperature-induced unfolding of rPrP in the absence of lipids (solid square) or presence of PE at following concentrations: 50 μM (black circles), 150 μM (black triangle), 250 μM (white triangels) or 500 μM (white diamonds). The data were normalized and the solid lines represent the nonlinear least-square fit of the data to a two-state unfolding model. (C) Temperature-induced unfolding of rPrP (20 μM) in the absence of lipids (square) or presence of 50 μM POPG (triangle). All experiments were performed in 20 mM Tris-HCl buffer, pH 7.5, 135 mM NaCl, 2 mM EDTA and 0.05% Triton. Temperature-induced unfolding was monitored by a circular dichroism at 222 nm.

To test whether rPrP secondary structure is affected by PE and POPG, CD spectra were collected for mixtures of rPrP with PE or POPG (Fig 2A). In the presence of POPG the CD signal and the characteristic signature of an α-helical conformation largely disappeared. These changes are indicative of POPG-induced unfolding of the α-helical domain and substantial rPrP aggregation that causes light scattering. In contrast to POPG, the α-helical conformation observed in the presence of PE was characterized by minor, if any, changes compared to that of rPrP alone (Fig 2A). This result illustrates that in contrast to POPG, PE does not significantly alter the secondary structure of rPrP.

To test further the effect of lipids on rPrP physical properties, we examined conformational stability of rPrP using temperature-induced denaturation. The temperature-induced unfolding profiles collected in the presence and absence of PE were superimposable showing very similar transition slopes and apparent Tm values (Fig 2B, Table 2). An increase in PE to rPrP molar ratio did not change the shape or position of the denaturation curves (Fig 2B). CD analysis with PE concentration above 500 μM was not possible due to high light scattering of PE. Nevertheless, the lack of effects on conformational stability and cooperativity of unfolding argues against strong physical interactions between PE and rPrP. In contrast to PE, a mixture of POPG and rPrP did not show cooperative transition upon heating (Fig 2C). Lack of cooperative transition supports previous observation that the α-helical domain is unfolded in the presence of POPG.

**Table 2:** Melting temperature of rPrP denaturation in the presence of PE.

Sample	apparent Tm, °C
rPrP	68.5±0.3
rPrP+50 μM PE	68.2±0.3
rPrP+150 μM PE	68.2±0.3
rPrP+250 μM PE	68.5±0.2
rPrP+500 μM PE	69.7±0.5

**Fig 3:**
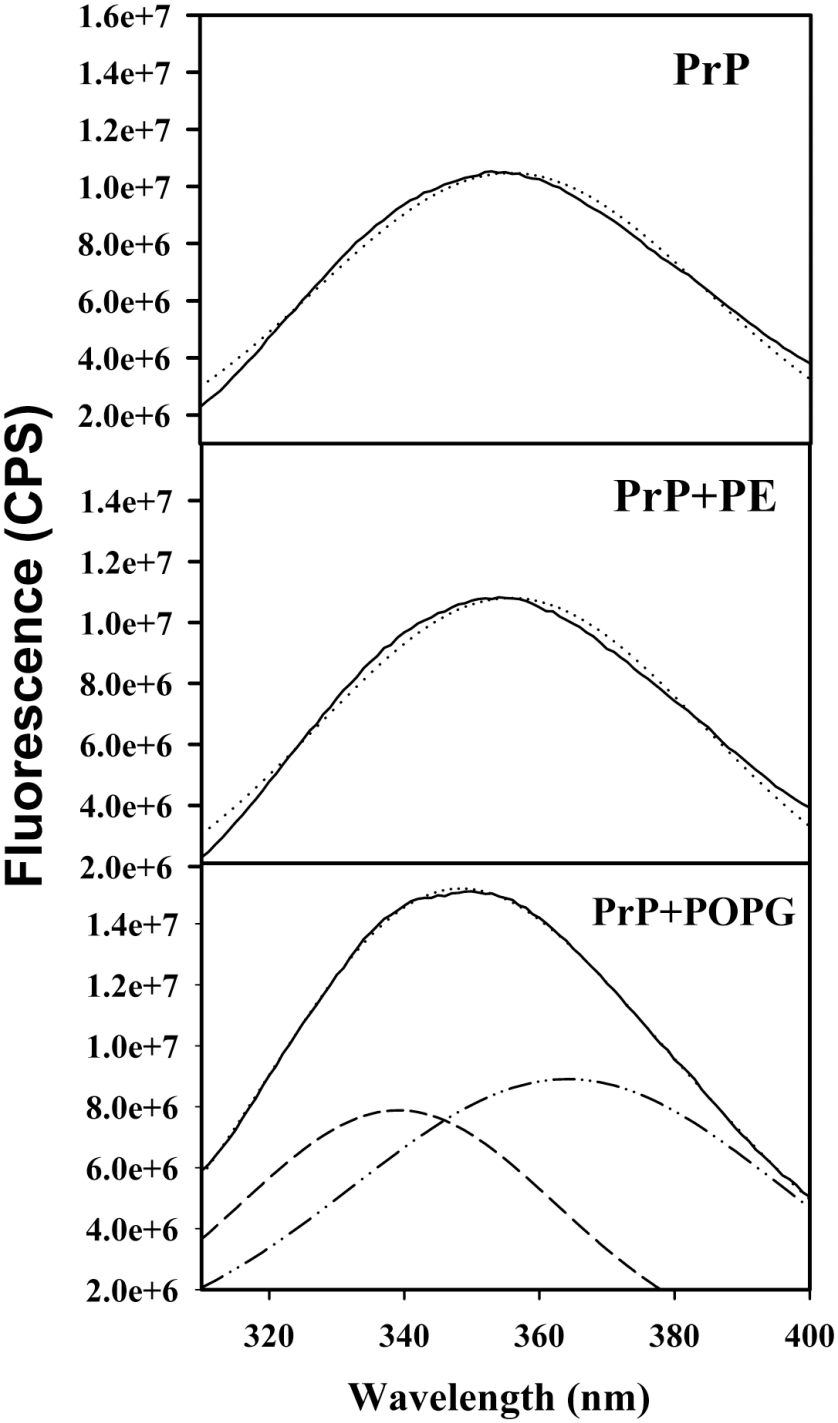
Analysis of tryptophan emission for assessing solvent environment. Fluorescent emission spectra collected for 10 μM rPrP alone (upper panel) or in the presence of 20 μM PE (middle panel) or 20 μM POPG (lower panel). In upper and middle panel, single Gaussian components are represented by dotted lines. In lower panel, individual Gaussian components are represented by dashed lines and their sum is represented by dotted line. All experiments were performed in 20 mM Tris-HCl buffer, pH 7.5, 135 mM NaCl, 2 mM EDTA and 0.05% Triton.

To test whether immediate solvent environment of rPrP changes in the presence of lipids, we collected tryptophan emission spectra. In rPrP the majority of tryptophan residues are located within the N-terminal region, which is unfolded in the monomeric α-rPrP. In rPrP alone or in the presence of PE, tryptophan residues displayed a single emission maximum at 354 nm (Fig 3 upper and middle panels), which is typical for unfolded protein regions exposed to a polar environment. However, in the presence of POPG a second emission maximum appeared at 340 nm in addition to the maximum at 354 nm (Fig 3 lower panel). While the emission spectra of rPrP alone or rPrP in the presence of PE could be fitted reasonably well using a single Gaussian component, two Gaussian components were required for fitting the spectra of rPrP in the presence of POPG (Fig 3). A blue shift in tryptophan fluorescence suggests a change in solvent environment toward more hydrophobic. A double maxima in the presence of POPG suggests that at least a fraction of tryptophan residues moved to a hydrophobic environment.

### Analysis of lipid-induced changes in rPrP hydrodynamic radius

**Fig 4:**
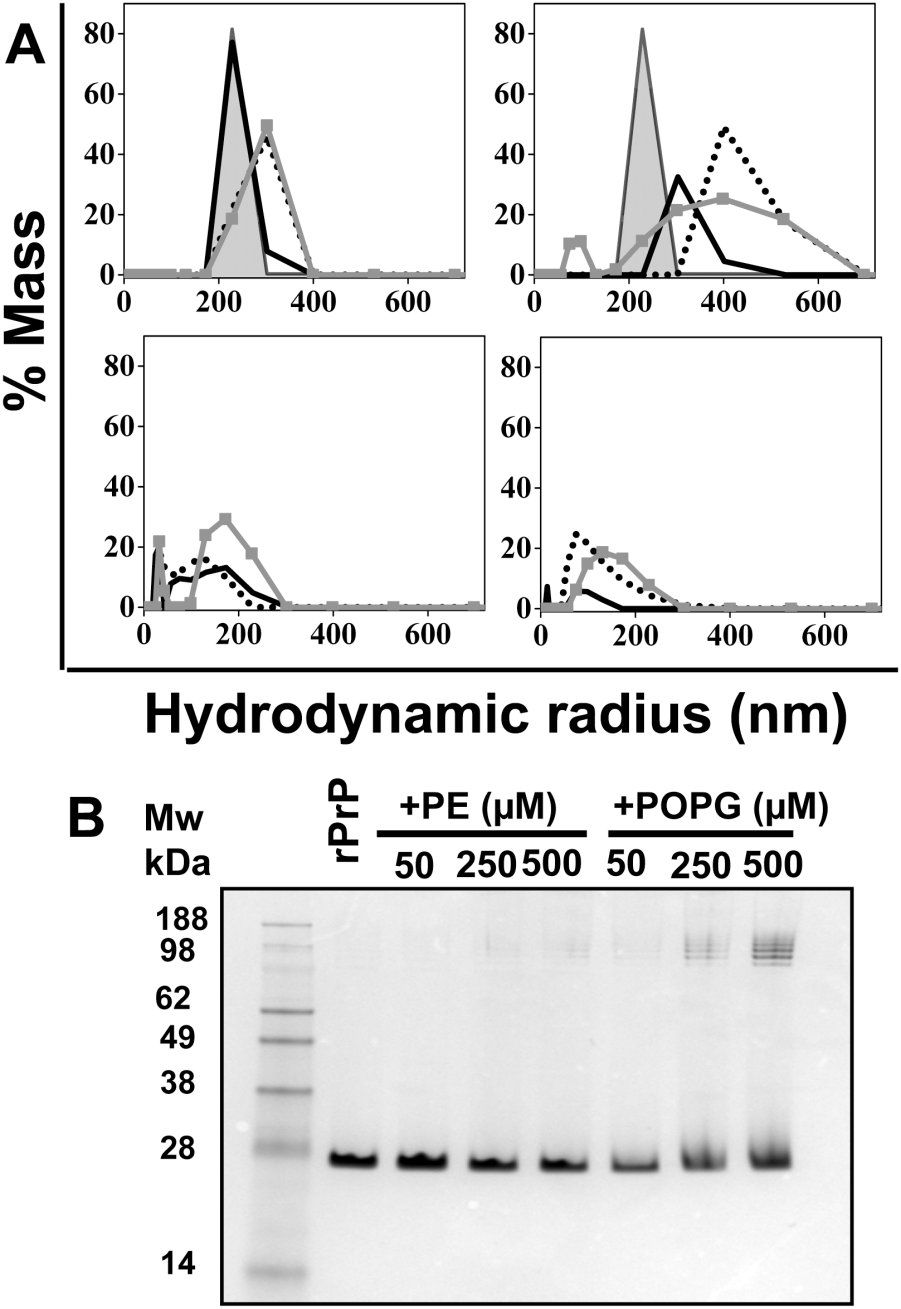
Analysis of rPrP hydrodynanic radius by dynamic light scattering. (A) Hydrodynamic radius of rPrP (2 μM) in the presence of PE (upper left panel) or POPG (upper right panel), or PE and POPG vesicles in the absence of rPrP (lower left and right panels, respectively). Hydrodynamic radius of rPrP without lipids is represented by filled grey area; rPrP in the presence of the following concentrations of PE or POPG: 2 μM by solid black lines, 10 μM by dotted lines, or 30 μM by grey lines with squares. (B) Analysis of lipid-induced rPrP aggregation by SDS-PAGE electrophoresis. rPrP (2 μM) was incubated for 16 hours with PE or POPG at indicated concentrations and analyzed by SDS-PAGE. Samples were mixed with a loading buffer in the absence of SDS at room temperature and then loaded to SDS-PAGE without heat denaturation step. All experiments in panels A and B were performed in 20 mM Tris-HCl buffer, pH 7.5, 135 mM NaCl, 2 mM EDTA and 0.05% Triton.

To test whether lipids change rPrP aggregation state, the hydrodynamic radius was analyzed using dynamic light scattering. Very modest increment in an average hydrodynamic radius was observed upon addition of 2, 10 or 30 μM PE to rPrP (Fig 4A). However, similar increment in the hydrodynamic radius was also observed for PE vesicles alone in the absence of rPrP, an effect attributable to an increase in size of the PE vesicles (Fig 4A). Supplementing rPrP with POPG within the same concentration range (2, 10 and 30 μM) induced substantially more profound increment in hydrodynamic radius (Fig 4A). In the absence of rPrP the hydrodynamic radius of POPG vesicles was substantially smaller than those found in the presence of the protein. Therefore, the observed increment in the hydrodynamic radius in rPrP/POPG solutions was attributable to formation of rPrP-POPG complexes.

To examine the strength of lipid-protein complexes, rPrP was incubated with PE or POPG and subjected to electrophoresis using SDS-PAGE. Upon incubation with PE, rPrP appeared largely as a monomer with very minor amounts of oligomers if any at a high molecular weight range (Fig 4B). This result suggests that if protein-protein or protein-lipid complexes are formed in the presence of PE, the intermolecular interactions are very weak. In contrast to PE, the presence of POPG produced a cluster of bands at high molecular weight range (Fig 4B). In addition, the band corresponding to monomeric rPrP displayed an upward smear, which was presumably due to strong rPrP-POPG interaction. This result is consistent with previous observations that, in contrast to PE, POPG binds to rPrP. This result also indicates that POPG-rPrP interaction is strong enough to resist, at least in part, the partially denaturing conditions.

### Analysis of rPrP interaction with lipids using sucrose density gradient centrifugation

**Fig 5:**
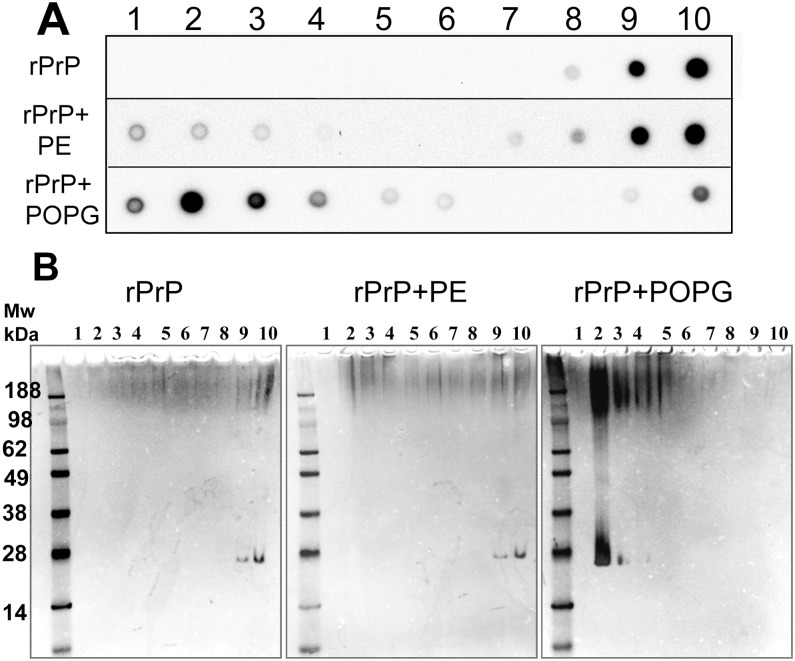
Analysis of interaction of rPrP with lipids by sucrose density gradient. (A) Distribution of rPrP in sucrose gradient fractions in samples containing rPrP only, or rPrP in the presence of 500 μM PE or 500 μM POPG as analyzed by dot blot. rPrP was incubated with PE or POPG in 20 mM Tris-HCl buffer, pH 7.5, 135 mM NaCl, 2 mM EDTA and 0.05% Triton for 16 hours at room temperature before centrifugation. Sucrose gradients were prepared using the same buffer solution. Fraction # 1 has the lowest density, and fraction # 10 has the highest density. (B) Analysis of sucrose gradient fractions from the panel A using SDS-PAGE followed by silver staining. Samples were mixed with a loading buffer in the absence of SDS at room temperature and then loaded to SDS-PAGE without heat denaturation step.

To estimate the fraction of rPrP that binds to lipid vesicles, sucrose density gradient centrifugations were performed using conditions that separate unbound proteins from lipid vesicles (16 h at 140,000 *g*). As expected, in the absence of lipids the majority of rPrP was found in fractions # 9 and 10 that contained high-density particles (Fig 5A). In the presence of PE, the majority of rPrP was again found in high-density fractions (# 9 and 10), whereas small amounts of protein was observed in low-density fractions # 1, 2, 3. In the presence of POPG, rPrP distribution was opposite. The majority of rPrP was found in low-density fractions # 2 and 3, whereas minor amounts were in high-density fraction # 10 (Fig 5A). This experiment illustrated that in the presence of POPG and PE rPrP exhibit opposite preferences for high and low density fractions.

To examine an aggregation state of rPrP, the materials from the high and low-density fractions was subjected to SDS-PAGE gels (Fig 5B). SDS-PAGE confirmed the effect of lipids on distribution of rPrP between sucrose gradient fractions assayed by dot blot. In the absence of lipids and in the presence of PE rPrP was detected in high-density fractions 9 and 10, whereas in the presence of POPG the majority of rPrP was found in low-density fractions # 2 and 3 (Fig 5B). More important, in the presence of POPG the majority of protein in fractions # 2 and 3 was found in highly aggregated states that were visible as smeared bands in high molecular weight region (Fig 5B). This experiment indicates that interaction between POPG and rPrP resulted in formation of aggregated protein-lipid complexes of low-density that are stable under partially denaturing conditions of SDS-PAGE gels.

## Discussion

In recent studies, two experimental protocols employed two different synthetic lipids PE and POPG for generating PrP^Sc^ using rPrP as a substrate [24, 25]. Because physical properties of PE and POPG are different, the question of whether the mechanism of lipid-assisted prion replication is common for different lipids is of great interest. The current work revealed that under the solvent conditions used for the conversion of rPrP into PrP^Sc^ in PMCA, POPG and PE exhibited drastically contrasting effects on the physical properties of rPrP.

We found that PE did not have any notable effects of on a secondary structure, thermodynamic stability or cooperativity of unfolding of the α-helical rPrP domain. In contrast, in the presence of POPG rPrP lost its α-helical secondary structure as evident from CD analysis (Figs 1 and 2). As judged from tryptophan fluorescence emission, no changes in immediate solvent environment were detected in the presence of PE, whereas blue shift in the emission spectrum was observed in the presence of POPG (Fig 3). This points out to a shift in the rPrP solvent environment from polar to hydrophobic. Taken together these results argue against strong binding or association between PE and rPrP. Consistent with these results, the experiments on sucrose density gradient revealed that the presence of PE the majority of rPrP was observed in high-density fractions, in a manner similar to that of rPrP samples that lacked lipids. In contrast, in the presence of POPG the majority of rPrP was found in low-density fractions, where lipid vesicles typically float (Fig 5). This result argues that rPrP binds to lipid vesicles and that this binding is likely to result in a loss of α-helical structure. In addition to binding to POPG, distinct molecular packing of POPG-associated and free rPrP could contribute to the differences in distribution of rPrP in the presence of PE and POPG. As judged from dynamic light scattering, POPG-rPrP complexes were several hundred nm in size (Fig 4). Notably, resistance of rPrP-POPG aggregates to partially denaturing conditions of SDS-PAGE gels illustrates the strength of interaction between rPrP and POPG (Fig 4B).

While involvement of lipids as cofactor of prion replication was demonstrated relatively recently, the study of interaction of PrP^Sc^ or rPrP with lipids has a long history. Solubilization of PrP^Sc^ using phospholipids leading to liposome-protein complexes was found to result in a significant increase in prion infectivity [16]. Consistent with the current study, previous work on rPrP-lipid interaction revealed that anionic lipid phosphatidylserine interacts with rPrP and destabilizes its α-helical domain [23]. The α-helical domain 90–231 was also found to have a high affinity for another negatively charged lipid POPG, but did not bind to zwitterionic lipids palmitoyloleoylphosphatidylcholine 1-palmitoyl-2-oleoyl-*sn*-glycero-3-phosphocholine (POPC) or dioleoylphosphatidylcholine(1,2-dioleoyl-*sn*-glycero-3-phosphocholine) (DOPC) [17, 19]. Highly basic pI of rPrP, which is above 9.0, explains high affinities of rPrP binding to anionic lipids. Indeed, electrostatic and hydrophobic interactions were shown to be involved in stabilizing complexes of rPrP with anionic lipids [19]. Binding of α-helical rPrP(90–231) to POPG was shown to promote β -sheet rich conformation and aggregation of in rPrP(90–231) and disrupt an integrity of a lipid bilayer. [17, 18]. While we observed POPG-induced loss of α-helical conformation and aggregation, we did not detect β-sheet rich structure in the presence of POPG. These differences could be due to differences in solvent conditions or rPrP size (full-length versus truncated rPrPs) employed in the current and previous studies, respectively. Composition of a lipid membrane, solvent conditions including pH and ionic strength, and the initial conformation of rPrP were found to be important in determining the strength of binding of rPrP to lipids and the effect of lipids on rPrP conformation [32, 33].

Consistent with previous studies [31, 34], we observed that rPrP was a monomer at pH 5 and low ionic strength, but formed aggregates at solvent conditions used for PMCA (pH 7.5, 135 mM NaCl) even in the absence of lipids. While rPrP aggregation at neutral pH occurred without notable changes in protein conformation, which remained predominantly α-helical, the conformational stability of the α-helical domain decreased significantly. Similar changes in conformational stability and aggregation were also observed with an increase in protein concentration (Fig 1). Aggregation of α-rPrP was accompanied by a loss of conformational stability regardless of whether changes of protein concentration or pH were the underlying causes of aggregation. These results point out that solvation of the surface area of the native state contribute positively to the thermodynamic stability, whereas protein-to-protein intermolecular contacts within the aggregated states counteract in part this positive contribution.

The result of the current work is consistent with the previous studies that examined interaction of POPG with rPrP. The study that employed iodixanol density gradient and several rPrP variants with deleted regions identified two regions important for POPG-rPrP interaction: the N-terminal positively charged region and the highly conserved central domain that consists of positively charged and hydrophobic amino acid regions [22]. In agreement with the current results, application of deuterium exchange mass spectrometry revealed that incubation of rPrP with POPG induced major conformational changes in rPrP that involved significant increase in accessibility of all three α helices and one β strand within the α-helical domain [35]. Consistent with the current data on dynamic light scattering, negative-stain electron microscopy revealed presence of amorphous aggregates of several hundreds nanometer in size in rPrP-POPG mixtures [36]. Interestingly, while POPG induced major conformational change in rPrP, solid-state NMR measurements found no evidence that POPG incorporates into advanced rPrP conversion products suggesting that POPG-rPrP complexes might form only at the initial stages of conversion [36].

The current work revealed that the mechanisms by which lipids assist prion replication might be fundamentally different for POPG and PE. POPG interacts directly with rPrP. One can speculate that rPrP-POPG interactions result in unfolding of rPrP that reduces the energy barrier of the conversion reaction. In addition, formation of POPG/rPrP complexes could also increase the local concentration of the protein facilitating the conversion. It is also possible that while POPG vesicles sequester the majority of rPrP, rPrP conversion does not occur directly through unstructured state associated with POPG vesicles, but through a state released by lipid vesicles. The last mechanism is consistent with the results of NMR study in which POPG was not detected in rPrP conversion products [36].

In the absence of any significant effect of PE on rPrP physical properties, it is difficult to propose the mechanism by which PE assists rPrP conversion. No indications of strong binding of PE with rPrP were observed. It is possible that PE-rPrP interaction is so weak and/or transient (PE-rPrP complexes exists for very short time periods) that it cannot be detected by steady-state techniques employed in the current study. If this is true, only a tiny fraction of rPrP can be found in a state bound to PE at any given time, the fraction that could be presumably an intermediate toward PrP^Sc^. Alternatively, PE might assist rPrP conversion into infectious states indirectly—by binding and neutralizing intermediates toward alternative, non-infectious amyloid states. A third possibility is that PE is involved transiently at the stage of interaction of rPrP with PrP^Sc^ seed. While seeds were not required for generating prions in the presence of POPG [24], the PMCA reactions where PE was employed were seeded with PrP^Sc^ [25, 27]. It is not clear whether multiple mechanisms by which lipids assist conversion of the prion protein *in vitro* also take place in a cell.
